# Blastocyst Morphology Holds Clues Concerning The Chromosomal Status of The Embryo 

**DOI:** 10.22074/ijfs.2015.4242

**Published:** 2015-07-27

**Authors:** Rita de Cassia Savio Figueira, Amanda Souza Setti, Daniela Paes Almeida Ferreira Braga, Assumpto Iaconelli Jr., Edson Borges Jr.

**Affiliations:** 1 Fertility -Assisted Fertilization Center, Sao Paulo -SP, Brazil; 2 Institute Sapientiae -Educational and Research Center in Assisted Reproduction, Sao Paulo -SP, Brazil

**Keywords:** Aneuploidy, Dysmorphism, *In Vitro* Fertilization, Preimplantation Genetic Screening

## Abstract

**Background:**

Embryo morphology has been proposed as an alternative marker of chro- mosomal status. The objective of this retrospective cohort study was to investigate the association between the chromosomal status on day 3 of embryo development and blas- tocyst morphology.

**Materials and Methods:**

A total of 596 embryos obtained from 106 cycles of intra- cytoplasmic sperm injection (ICSI) followed by preimplantation genetic aneuploidy screening (PGS) were included in this retrospective study. We evaluated the relation- ship between blastocyst morphological features and embryonic chromosomal altera- tion.

**Results:**

Of the 564 embryos with fluorescent in situ hybridization (FISH) results, 200 reached the blastocyst stage on day 5 of development. There was a significantly high- er proportion of euploid embryos in those that achieved the blastocyst stage (59.0%) compared to embryos that did not develop to blastocysts (41.2%) on day 5 (P<0.001). Regarding blastocyst morphology, we observed that all embryos that had an abnormal inner cell mass (ICM) were aneuploid. Embryos with morphologically normal ICM had a significantly higher euploidy rate (62.1%, P<0.001). As regards to the trophectoderm (TE) morphology, an increased rate of euploidy was observed in embryos that had nor- mal TE (65.8%) compared to embryos with abnormal TE (37.5%, P<0.001). Finally, we observed a two-fold increase in the euploidy rate in high-quality blastocysts with both high-quality ICM and TE (70.4%) compared to that found in low-quality blastocysts (31.0%, P<0.001).

**Conclusion:**

Chromosomal abnormalities do not impair embryo development as ane- uploidy is frequently observed in embryos that reach the blastocyst stage. A high-quality blastocyst does not represent euploidy of chromosomes 13, 14, 15, 16, 18, 21, 22, X and Y. However, aneuploidy is associated with abnormalities in the ICM morphology. Further studies are necessary to confirm whether or not the transfer of blastocysts with low-quality ICM should be avoided.

## Introduction

In order to maximize the success rates of assisted reproductive techniques (ART), a reliable means of identifying the embryo with the best prognosis and the highest potential for implantation is required. Because of the high frequency of aneuploid embryos and the negative outcomes associated with their transfer, the identification and transfer of chromosomally normal embryos is of pivotal importance, thus increasing the likelihood that the embryos are viable, leading to improved implantation and pregnancy rates, and reduced miscarriage rates (1).

Embryo morphology has been proposed as an alternative marker of chromosomal status (1, 2). Some studies suggest a link exists between the distribution and number of nucleoli in the pronuclei and the chromosomal status of the zygote (3, 4). In addition, it has been found that arrested cleavagestage embryos, as well as embryos that present with abnormal rates of cleavage, exhibited a high frequency of chromosomal abnormalities (5).

Other studies that have searched for a link between aneuploidy and altered embryo morphology (6, 7) suggested that morphology could be a useful indicator of aneuploidy in some embryos and under some conditions. Therefore, the aim of this study was to investigate the association between the chromosomal status of the embryo on day 3 of development and blastocyst morphology.

## Materials and Methods

Experimental design, patients and inclusion criteria

Using our centre’s computerized database we retrospectively identified 106 cycles, performed from January 2010 to December 2010, which fulfilled the following inclusion criteria: intracytoplasmic sperm injection (ICSI) followed by preimplantation genetic aneuploidy screening (PGS). The indications for chromosome screening were advanced reproductive age (>35 years), history of unsuccessful *in vitro* fertilization (IVF) attempts and/or miscarriages. To minimize the influence of male factor infertility, all cases of sperm concentration less than 1×106 M/mL and sperm motility less than 20% were excluded from the study. The relationship between blastocyst morphological features and embryonic chromosomal alteration was evaluated.

Written informed consent was obtained, in which patients agreed to share the outcomes of their own cycles for research purposes, and the study was approved by the local Institutional Review Board.

### Controlled ovarian stimulation, oocytes and embryo culture

The stimulation protocol, preparation of oocytes and embryo culture were described elsewhere (8). Full blastocysts onwards, presenting morphologically normal inner cell mass (ICM) and trophectoderm (TE) were defined as high-quality blastocysts. A tightly packed ICM that contained numerous cells was defined as a high quality ICM. Similarly, the TE was classified as high quality by the presence of numerous cells forming a cohesive epithelium (9).

### Embryo biopsy

Embryos that reached at least the 5-cell stage on day 3 of development were biopsied by laser zona drilling using a 1.48 μm Infrared Diode Laser (Octax Laser Shot System, MTG, Bruckberg, Germany) and returned to culture. Only one blastomere was removed per embryo. The definition of a successful biopsy was the removal of a cell without lysis, so that the cell could be used for fixation and analysis.

### Blastomere fixation and fluorescent in situ hybridization (FISH)

The blastomere of an embryo was fixed on a slide using the HCI/Tween 20 method as previously described (10). A two-round fluorescent in situ hybridization (FISH) procedure was performed which allowed for the detection of chromosomes X, Y, 13, 18 and 21 (Multivision PGT Probe Panel; Vysis, Downers Grove, IL, USA) in the first round and chromosomes 14, 15, 16 and 22 in the second round. The hybridization solution for the second round was prepared by mixing a probe for chromosome 14 (Vysis, Telvysion 14q/D14S1420 probe, Spectrum Orange), 15 (Vysis, Telvysion 15q/D15Z1, Spectrum Aqua), 16 (Vysis, Satellite II DNA/D16Z3 probe, Spectrum Orange) and 22 (Vysis, LSI 22, 22q11.2, Spectrum Green). The results were analyzed using a fluorescence microscope.

### Fluorescent in situ hybridization scoring criteria

At diagnosis, we considered embryos as normal when two sex chromosomes and two chromosomes (13, 14, 15, 16, 18, 21 and 22) were present. They were considered trisomic or monosomic, respectively, if an extra or missing signal was observed. Finally, we defined embryos as haploid, triploid or polyploid if one, three or more copies, respectively, of the set of chromosomes were present. The presence of two or more chromosomal abnormalities within the same blastomere was characterized as multiple abnormalities.

### Embryo transfer

Embryo transfer was performed on day 5 of development using a soft catheter. One to three euploid embryos were transferred per patient.

### Clinical follow-up

A pregnancy test was performed 12 days after embryo transfer. A positive pregnancy test confirmed biochemical pregnancy. All women with a positive test had a transvaginal ultrasound scan 2 weeks after the positive test, a clinical pregnancy was diagnosed when the fetal heartbeat was detected. Pregnancy rates were calculated per transfer. Miscarriage was defined as pregnancy loss before 20 weeks.

### Statistical analysis

We compared the incidence of euploid and aneuploid embryos according to the morphologic characteristics of the embryo on day 5 of development. Qualitative variables were compared using the chi-square or Fisher’s exact tests. The influence of chromosomal constitution on the blastocyst morphology was investigated through binary logistic regression, adjusted for maternal age. The results were expressed as odds ratio (OR), confidence intervals (CI) and P values. Results were considered to be significant at P<0.05. Statistical analysis was carried out using MINITAB 16 Software.

## Results

The general characteristics of the cycles are shown in table 1. The mean ± SD female age was 37.0 ± 4.7 years (range: 25–46 years). Of 106 cycles started, 90 were transferred (84.9%). The implantation rate was 26.7%, pregnancy rate was 28.9% and no miscarriage occurred for any of the patients who became pregnant.

**Table 1:** General characteristics of the intracytoplasmic sperm injection (ICSI) cycles


Variable	Value

Female age (Y)	37.0 ± 4.7
Male age (Y)	40.8 ± 6.7
FSH (IU)	2448.6 ± 641.6
E_2_ (pg/mL)	2220.0 ± 1461.0
Follicles (n)	18.0 ± 11.9
Oocytes (n)	13.2 ± 8.7
MII oocytes (n)	10.4 ± 7.4
MII oocyte rate (%)	78.8
Injected oocytes (n)	10.5 ± 6.8
Fertilization rate (%)	75.6
High-quality embryo rate (%)	70.7
Transferred embryos (n)	1.3
Transferred cycles (%)	90/106 (84.9)
Implantation rate/	31/117 (26.5)
transferred embryos (%)	
Pregnancy/transferred cycle (%)	26/90 (28.9)
Miscarriage/pregnancy (%)	0/26 (0.0)


FSH; Follicle-stimulating hormone, E_2_: Estradiol and MII; Metaphase II.

Out of 596 embryos successfully biopsied on day 3 of development, 564 had FISH results. An inconclusive diagnosis was obtained in 32 (5.4%) cells due to technical issues that included hybridization failure, signal overlapping yielding false-negative results, and split or diffuse signals. A total of 240 embryos were euploid (42.6%) and 324 were aneuploid (57.4%). The detailed distribution of aneuploidy is shown in table 2.

**Table 2:** Distribution of aneuploidy in embryos on day 3 of development


Type of abnormality	Affected embryos (%)	Affected chromosomes								
		13	14	15	16	18	21	22	X	Y

Multiple	107/324 (33.0)	56	0	18	0	56	70	0	39	4
Mosaic	2/324 (0.6)	0	0	0	0	0	2	0	0	0
Monosomy	96/324 (29.6)	28	2	4	4	20	16	0	20	2
Trisomy	119/324 (36.7)	36	2	2	18	14	39	4	4	0


Note: Columns 3-11 represent number of embryos with the respective chromosome affected.

Of the 564 embryos with FISH results, 200 reached the blastocyst stage on day 5 of development (35.5%). A total of 118 blastocysts were euploid (59.0%) and 82 were aneuploid (41.0%) on day 3 of development.

There was a significantly higher proportion of euploid embryos in those that achieved the blastocyst stage (59.0%) compared to embryos that did not develop to a blastocyst on day 5 (41.2%, P<0.001).

**Fig.1:**
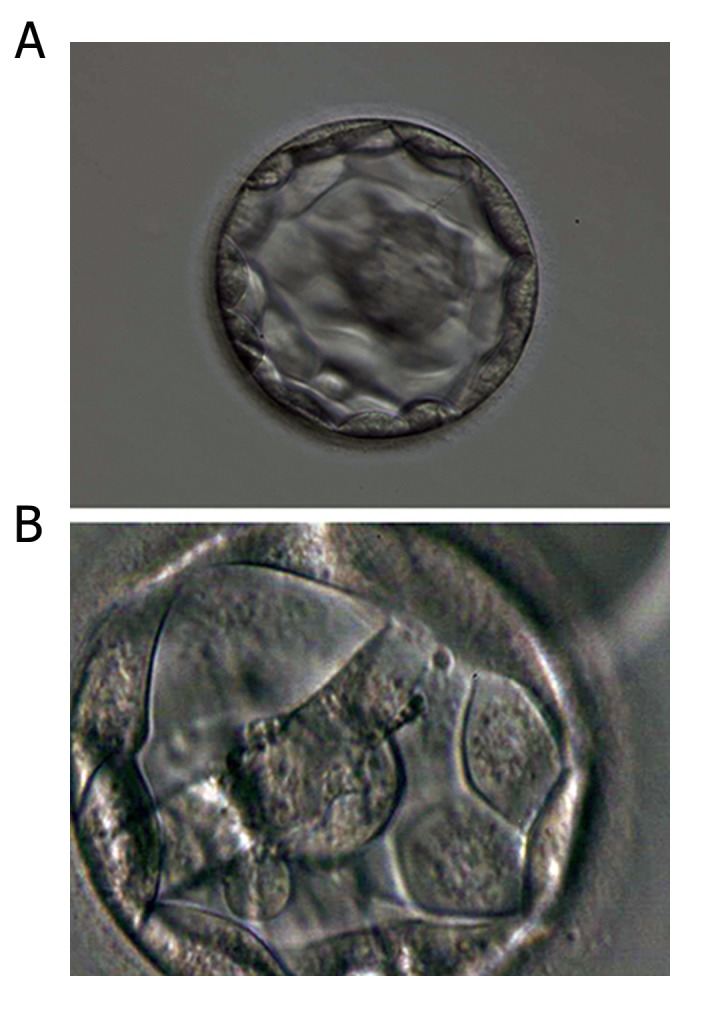
Blastocysts showing high- and low-quality inner cell mass (ICM) and trophectoderm (TE). A. A high-quality blastocyst showing a normal ICM with many cells that are tightly compacted, and a normal TE with many cells that form a cohesive epithelium lining the blastocoel cavity. B. A low-quality blastocyst showing an abnormal ICM that is loosely made up of only a few cells. Large TE cells that stretch over great distances to reach the next cell.

In terms of blastocyst morphology, we observed that all embryos with abnormal ICM were aneuploid. There was a significantly higher euploidy rate in embryos with a morphologically normal ICM (62.1%, P<0.001). An increased rate of euploidy was observed in embryos that showed normal TE (65.8%) compared embryos with abnormal TE (37.5%, P<0.001). Finally, we observed a 2-fold increase in the euploidy rate in high-quality blastocysts that had both high-quality ICM and TE (70.4%) compared to low-quality blastocysts (31.0%, P<0.001, Fig.1, Table 3).

The results of the logistic regression models demonstrated an increase in the probability of euploidy when: i. embryos reached the blastocyst stage on day 5 of development (OR: 2.09, CI: 1.29–3.39, P=0.002), ii. blastocysts showed normal TE (OR: 3.21, CI: 1.24–8.31, P=0.015) and iii. blastocysts showed both normal TE and ICM (OR: 5.29, CI: 2.07–13.51, P<0.001).

Neither the presence of monosomies (OR: 1.77, CI: 0.87–3.59, P=0.113), nor the presence of trisomies (OR: 2.98, CI: 0.79–11.21, P=0.880) influenced blastocyst formation. However, the presence of multiple abnormalities negatively influenced the odds of development to the blastocyst stage (OR: 0.20, CI: 0.01–0.56, P=0.012). Finally, the percentage of euploid blastocysts did not influence implantation (Slope: 47.65, R2: 1.7%, P=0.413) or pregnancy (OR: 1.03, CI: 0.98–1.08, P=0.273) rates.

**Table 3:** Comparison of euploidy and aneuploidy rates according to blastocyst development and morphology


Predictor variables	Dependent variables	
	Euploidy (%)	Aneuploidy (%)

Embryo development on D5		
Blastocyst	118/200 (59.0)	82/200 (41.0)
Non-blastocyst	150/364 (41.2) ^a^	214/364 (58.8) ^a^
Blastocyst morphology		
ICM		
Normal	118/190 (62.1)	72/190 (37.9)
Abnormal	0/10 (0.0)^b^	10/10 (100) ^b^
TE		
Normal	100/152 (65.8)	52/152 (34.2)
Abnormal	18/48 (37.5) ^c^	30/48 (62.5) ^c^
ICM+TE		
Normal	100/142 (70.4)	42/142 (29.6)
Abnormal	18/58 (31.0) ^d^	40/58 (69.0) ^d^


D5; Day five of development, ICM; Inner cell mass, TE; Trophectoderm, ^a^; Significantly different from blastocyst group, ^b^; Significantly different from normal ICM group, ^c^; Significantly different from normal TE group and ^d^: Significantly different from normal ICM+TE group.

## Discussion

The objective of this study was to investigate the relationship between blastocyst morphology and the chromosome status of embryos on day 3 of development. Our results demonstrated significant differences in the euploidy rate between embryos that achieved blastocyst stage on day 5 compared to embryos that did not. As for blastocyst morphology, we observed significant differences in the euploidy rate between the groups with i. normal and abnormal ICM, ii. normal and abnormal TE and iii. normal and abnormal ICM plus TE. The results of the logistic regression models demonstrated a 2-fold increase in the probability of euploidy when embryos reached the blastocyst stage on day 5 of development, a 3-fold increase in the probability of euploidy when blastocysts showed normal TE, and a 5-fold increase in the probability of euploidy when blastocysts showed both normal TE and ICM.

Previous studies have investigated the relationship between embryo morphology and aneuploidy. Although preliminary, these studies have shown a weak association between aneuploidy and abnormal embryo morphology. (2, 5-7, 11-13). Alfarawati et al. (2) showed that aneuploidy negatively affected the ICM and TE grades. Morphologically, poor blastocysts had a higher incidence of monosomy and abnormalities that affected several chromosomes. Magli et al. (5) observed that the incidence of chromosomal abnormalities was significantly higher in embryos that divided according to a time frame and a symmetry plan which were different from expected.

The main question is whether morphological analysis can be of assistance in the selection of euploid embryos for transfer. A recent study has shown that the aneuploidy rate observed on day 5 could be reduced from 56 to 48% if only embryos that achieved the top grades were selected for transfer (2). In addition, Munne et al. (6) showed an euploidy incidence of 44% in morphologically normal embryos and 30% in morphologically abnormal embryos. These results were consistent with the findings of the present study which showed that a high incidence of aneuploidy could be found in morphologically normal embryos.

This study showed a link between euploidy and normal blastocyst ICM and TE morphologies. We found increased euploidy rates amongst blastocysts with good ICM and TE morphology and a lower likelihood of euploidy in low-quality blastocysts. In light of these results we could suggest that blastocyst morphology might be a useful indicator of embryo chromosome constitution. This would be an attractive possibility, as chromosome assessment based upon morphology would allow embryo biopsy to be avoided, resulting in an inexpensive test with no impact on the embryo. However, as seen in the present study, it was important to note that over 40% of embryos which reached the blastocyst stage were aneuploid. Moreover, 35% of the blastocysts that presented with morphologically normal TE and approximately 30% of high-quality blastocysts were aneuploid. Therefore, the development to blastocyst and morphological normalcy of the ICM plus TE could not be used to predict euploidy for the chromosomes analyzed in this study. On the other hand, despite the observation that 38% of blastocysts with normal ICM were aneuploid, in this study all embryos that had abnormal ICM were aneuploid. Therefore, an abnormal ICM could predict aneuploidy for the chromosomes analyzed in this study. Nonetheless, of note, only 10 embryos showed low-quality ICM in the present study.

Our study possesses three drawbacks, as follows:

1. This is a retrospective study that lacks sample size calculation and therefore is subject to bias and underpowered results.
2. A single blastomere biopsy, which does not rule out the risk of embryo mosaicism, has been performed. Nevertheless, since no conclusive data has demonstrated the superiority of doubleover single-blastomere biopsy (14, 15), a single blastomere biopsy is routinely performed in our center.
3. We assessed a limited number of chromosomes frequently involved in term pregnancies with potentially severe clinical consequences. Therefore it was inevitable that some of the embryos categorized as euploid were in fact abnormal with aneuploidies that affected chromosomes which were not tested.

It has been suggested that blastocyst culture may select against aneuploidy (16); however, certain abnormalities are compatible with development to term. Despite evidence for improved selection with blastocyst culture, our data suggest that extended culture to the blastocyst stage does not definitively select for euploid embryos.

## Conclusion

Chromosomal abnormalities do not impair embryo development as aneuploidy is frequently observed in embryos that reach the blastocyst stage. High-quality blastocysts are not representative of euploidy of chromosomes 13, 14, 15, 16, 18, 21, 22, X and Y. However, aneuploidy is associated with abnormalities in the ICM morphology. Further studies are necessary to confirm whether or not we should avoid the transfer of blastocysts with low-quality ICM.

## References

[1] Wells D (2010). Embryo aneuploidy and the role of morphological and genetic screening. Reprod Biomed Online.

[2] Alfarawati S, Fragouli E, Colls P, Stevens J, Gutierrez- Mateo C, Schoolcraft WB (2011). The relationship between blastocyst morphology, chromosomal abnormality, and embryo gender. Fertil Steril.

[3] Balaban B, Yakin K, Urman B, Isiklar A, Tesarik J (2004). Pronuclear morphology predicts embryo development and chromosome constitution. Reprod Biomed Online.

[4] Gianaroli L, Magli MC, Ferraretti AP, Lappi M, Borghi E, Ermini B (2007). Oocyte euploidy, pronuclear zygote morphology and embryo chromosomal complement. Hum Reprod.

[5] Magli MC, Gianaroli L, Ferraretti AP, Lappi M, Ruberti A, Farfalli V (2007). Embryo morphology and development are dependent on the chromosomal complement. Fertil Steril.

[6] Munne S, Chen S, Colls P, Garrisi J, Zheng X, Cekleniak N (2007). Maternal age, morphology, development and chromosome abnormalities in over 6000 cleavage-stage embryos. Reprod Biomed Online.

[7] Staessen C, Van Steirteghem A (1998). The genetic constitution of multinuclear blastomeres and their derivative daughter blastomeres. Hum Reprod.

[8] Setti AS, Cortezzi SS, Figueira Rde C, Martinhago CD, Braga DP, Iaconelli A Jr (2012). A chromosome 19 locus positively influences the number of retrieved oocytes during stimulated cycles in Brazilian women. J Assist Reprod Genet.

[9] Gardner DK, Schoolcraft WB, Jansen R, Mortimer D (1999). In vitro culture of human blastocysts. Toward reproductive certainty: fertility and genetics beyond.

[10] Coonen E, Dumoulin JC, Ramaekers FC, Hopman AH (1994). Optimal preparation of preimplantation embryo interphase nuclei for analysis by fluorescence in-situ hybridization. Hum Reprod.

[11] Eaton JL, Hacker MR, Harris D, Thornton KL, Penzias AS (2009). Assessment of day-3 morphology and euploidy for individual chromosomes in embryos that develop to the blastocyst stage. Fertil Steril.

[12] Hardarson T, Caisander G, Sjogren A, Hanson C, Hamberger L, Lundin K (2003). A morphological and chromosomal study of blastocysts developing from morphologically suboptimal human pre-embryos compared with control blastocysts. Hum Reprod.

[13] Moayeri SE, Allen RB, Brewster WR, Kim MH, Porto M, Werlin LB (2008). Day-3 embryo morphology predicts euploidy among older subjects. Fertil Steril.

[14] Van de Velde H, De Vos A, Sermon K, Staessen C, De Rycke M, Van Assche E (2000). Embryo implantation after biopsy of one or two cells from cleavage-stage embryos with a view to preimplantation genetic diagnosis. Prenat Diagn.

[15] Edwards RG, Hansis C (2005). Initial differentiation of blastomeres in 4-cell human embryos and its significance for early embryogenesis and implantation. Reprod Biomed Online.

[16] Magli MC, Jones GM, Gras L, Gianaroli L, Korman I, Trounson AO (2000). Chromosome mosaicism in day 3 aneuploid embryos that develop to morphologically normal blastocysts in vitro. Hum Reprod.

